# Limits of Ligand Selectivity from Docking to Models: In Silico Screening for A_1_ Adenosine Receptor Antagonists

**DOI:** 10.1371/journal.pone.0049910

**Published:** 2012-11-21

**Authors:** Peter Kolb, Khai Phan, Zhan-Guo Gao, Adam C. Marko, Andrej Sali, Kenneth A. Jacobson

**Affiliations:** 1 Department of Pharmaceutical Chemistry, University of California San Francisco, San Francisco, California, United States of America; 2 Molecular Recognition Section, Laboratory of Bioorganic Chemistry, National Institute of Diabetes and Digestive and Kidney Diseases, National Institutes of Health, Bethesda, Maryland, United States of America; 3 Department of Bioengineering and Therapeutic Sciences, University of California San Francisco, San Francisco, California, United States of America; Medical School of Hannover, United States of America

## Abstract

G protein-coupled receptors (GPCRs) are attractive targets for pharmaceutical research. With the recent determination of several GPCR X-ray structures, the applicability of structure-based computational methods for ligand identification, such as docking, has increased. Yet, as only about 1% of GPCRs have a known structure, receptor homology modeling remains necessary. In order to investigate the usability of homology models and the inherent selectivity of a particular model in relation to close homologs, we constructed multiple homology models for the A_1_ adenosine receptor (A_1_AR) and docked ∼2.2 M lead-like compounds. High-ranking molecules were tested on the A_1_AR as well as the close homologs A_2A_AR and A_3_AR. While the screen yielded numerous potent and novel ligands (hit rate 21% and highest affinity of 400 nM), it delivered few selective compounds. Moreover, most compounds appeared in the top ranks of only one model. These findings have implications for future screens.

## Introduction

G protein-coupled receptors (GPCRs) are one of the pharmaceutically most important protein families, and the targets of around one third of present day drugs [1]. They mediate the transmission of signals from the exterior to the interior of a cell by binding signaling agents and, via conformational changes, eliciting intracellular responses. GPCRs consist of seven membrane-crossing helices. The binding pockets of the native small molecule ligands, i.e. orthosteric binding sites, are situated in the middle of the helical bundle in the Class A GPCR structures that have been determined so far [2]. Despite the recent advances in GPCR X-ray structure determination [3] and the substantial numbers of novel ligands identified for some GPCRs [4], [5], there are still many (potential) GPCR targets for which no structure or ligands are known. In order to apply protein structure-based methods of ligand identification, in particular docking, to receptors that lack an experimentally determined structure, homology modeling is a promising avenue. Constructing homology models is facilitated by the fact that the transmembrane (TM) region of Class A GPCRs is relatively well conserved [6]. The accuracy of homology models is limited, however, by the uncertainty of modeling the extra- and intracellular loops, which greatly vary in length and amino acid composition, even between otherwise closely related GPCRs [7].

In this study, we tested the utility of homology models for docking and selecting compounds with reasonable affinity for the investigated receptor subtype. We intentionally restricted the amount of existing ligand data used to refine the binding site during model building to mimic a situation where few ligands are known (as would be the case for previously little investigated “novel” targets). In fact, except for the very first steps of model building and optimization, only the affinity data obtained in this study was used to improve the homology models. Three sequential cycles of model refinement, docking, and ligand testing were applied, using the data acquired in previous rounds to guide the receptor model optimization in the following rounds. In parallel, we also probed the tendency of the screen to identify novel ligands of other subtypes within the same receptor family, i.e. the selectivity of a homology model-based screen against a *single* GPCR subtype. These findings were compared with the distribution of selectivity ratios of known ligands for the same subtypes.

**Figure 1:**
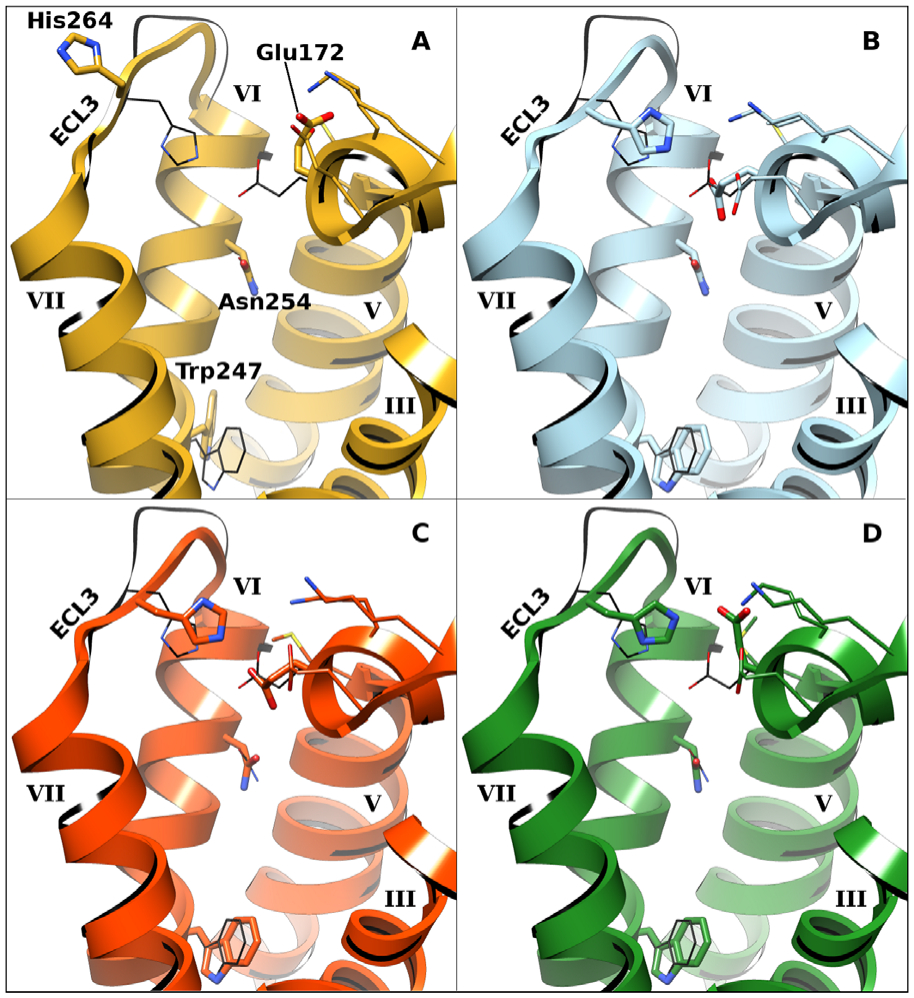
The four A_1_AR models used in this study. Helices are labeled with Roman numerals. For clarity, individual residues mentioned in the text, depicted as thick sticks, are only labeled in panel A. Additional residues that were optimized are in thin sticks, including Lys168^4.99^, Glu170, Lys173, and Met177. Helices I and II have been removed for clarity. The X-ray crystallographic structure of the A_2A_AR, the template (PDB 3EML), is shown in black.

The adenosine receptors (ARs), which consist of the four subtypes A_1_, A_2A_, A_2B_, and A_3_, have been chosen as a suitable test case for the application of virtual screening to a closely related subtype of a known GPCR structure. There are both antagonist-bound and agonist-bound X-ray structures known for the A_2A_AR subtype, with various ligands co-crystallized for each case. Thus, the region for orthosteric AR ligand binding has been well characterized. The first antagonist-bound structure to be determined was co-crystallized with the high affinity ligand 4-[2-[7-amino-2-(2-furyl)-1,2,4-triazolo[1,5-a] [1], [3], [5]triazin-5-yl-amino]ethylphenol (**1**, ZM241385, Fig. 4) [8], [9]. An unexpected orientation of the ligand perpendicular to the plane of the membrane bilayer was observed. Extracellular loops, as well as helical TM domains, are involved in coordinating the ligand. *In silico* virtual screening for A_2A_AR antagonists has already been demonstrated to be successful based on the inactive conformation of the A_2A_AR, as determined by crystallography [10], [49].

**Table 1:** In vitro affinity in binding to three subtypes of hARs of diverse heterocyclic derivatives identified through their high ranks in the *in silico* screen (structures are shown in Chart 2).

Compound ID	A_1_ a	A_2A_ a	A_3_ a	Model	Closest ChEMBL^b^
	*% Inhibition* * or K_i_ (nM)	*% Inhibition* * or K_i_ (nM)	*% Inhibition* * or K_i_ (nM)		
**7**	17±9%	**3310±270**	43±3%	**A**	0.53
**8**	**3460±420**	11±6%	35±4%	**A**	0.64
**9**	2±2%	**2360±260**	**4860±330**	**A**	0.47
**10**	9±9%	37±1%	**9060±1100**	**A**	0.57
**11**	13±9%	35±3%	**13,700±2200**	**A**	0.56
**12**	28±9%	**3655±870**	**2780±920**	**A**	0.72
**13**	10±10%	**10,900±2200**	**3480±1100**	**A**	0.60
**14**	19±10%	**6540±1090**	49±1%	**A**	0.25
**15**	20±4%	5±3%	**9330±1800**	**A**	0.30
**16**	13±2%	36±0.2%	**13,400±1900**	**A**	0.46
**17**	**400±60**	**740±390**	48±7%	**B**	0.49
**18**	**3430±1030**	**2130±720**	**1760±210**	**B**	0.41
**19**	**3340±560**	**6660±860**	23±3%	**B**	0.41
**20**	45%**	**3560±510**	**1520±360**	**B**	0.71
**21**	**980±90**	**1340±310**	**205±30**	**B**	0.39
**22**	36%**	**9300±700**	42±6%	**B**	0.32
**23**	**1220±340**	**3780±830**	**70±20**	**B**	0.50
**24**	33±9%	**6140±1690**	**40±6**	**D**	0.42
**25**	**2930±480**	**1450±170**	**550±70**	**D**	0.30
**26**	**3940±390**	**1370±470**	**3850±590**	**D**	0.27

a Binding in membranes of CHO (A_1_ and A_3_ARs) or HEK293 (A_2A_AR) cells stably expressing a hAR subtype. Total and nonspecific binding at the A_1_AR determined using [^3^H]DPCPX in the absence and presence of 10 µM CGS15943 (*N*-[9-chloro-2-(2-furanyl) [1], [2], [4]triazolo[1,5-c]quinazolin-5-amine), respectively.

b ECFP4 Tanimoto similarity for the most structurally similar known AR ligand (Table S3).

* percent inhibition at 10 µM compound concentration.

** n = 1.

Among the different subtypes, the A_1_AR is also an attractive pharmaceutical target. Its antagonists have been explored as kidney-protective agents, compounds for treating cardiac failure, cognitive enhancers, and antiasthmatic agents [11], [12]. Structurally diverse antagonists, such as the pyrazolopyridine derivative **2** and the 7-deazaadenine derivative **3**, were previously identified, and some of these compounds were under consideration for clinical use [13], [14]. The prototypical AR antagonists, i.e. the 1,3-dialkylxanthines, have provided numerous high affinity antagonists with selectivity for the A_1_AR. One such antagonist, rolofylline **4**, an alkylxanthine derivative of nanomolar affinity, was previously in clinical trials for cardiac failure [15].

**Figure 2:**
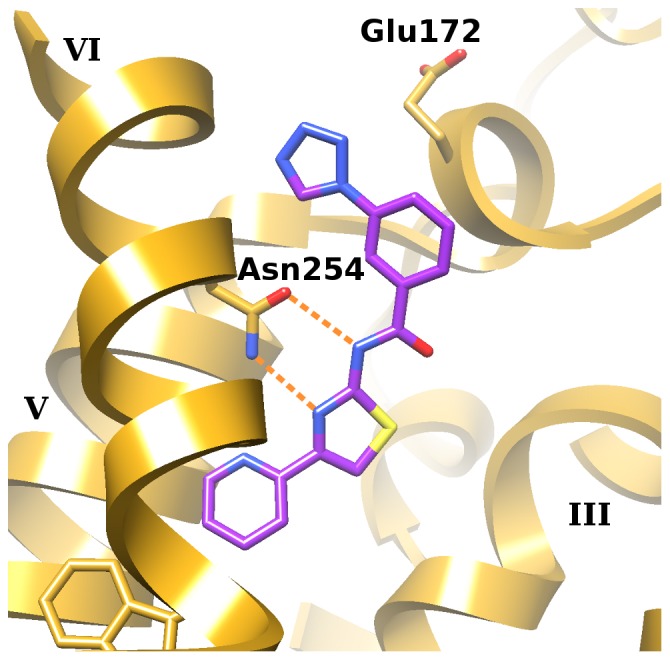
Calculated binding mode of compound 8, the ligand hit with the highest selectivity towards A_1_AR. The protein is model **A**. Orange dotted lines denote hydrogen bonds formed with Asn254^6.55^. Helices are labeled with roman numerals.

**Table 2:** Performance of the four homology models against the three AR subtypes.

	A_1_		A_2A_		A_3_		
MODEL	A/T^a^	%	A/T	%	A/T	%	Round
**A**	1/15	7%	5/15	33%	7/15	47%	1
**B**	5/12	42%	7/12	58%	4/12	33%	2
**C**	0/6	0%	0/6	0%	0/6	0%	3
**D**	2/6	33%	3/6	50%	3/6	50%	3
**S** b	8/39	21%	15/39	38%	14/39	36%	

a number of actives (A) over number of molecules tested (T).

b sum: overall hit rate for all tested ligands.

**Figure 3:**
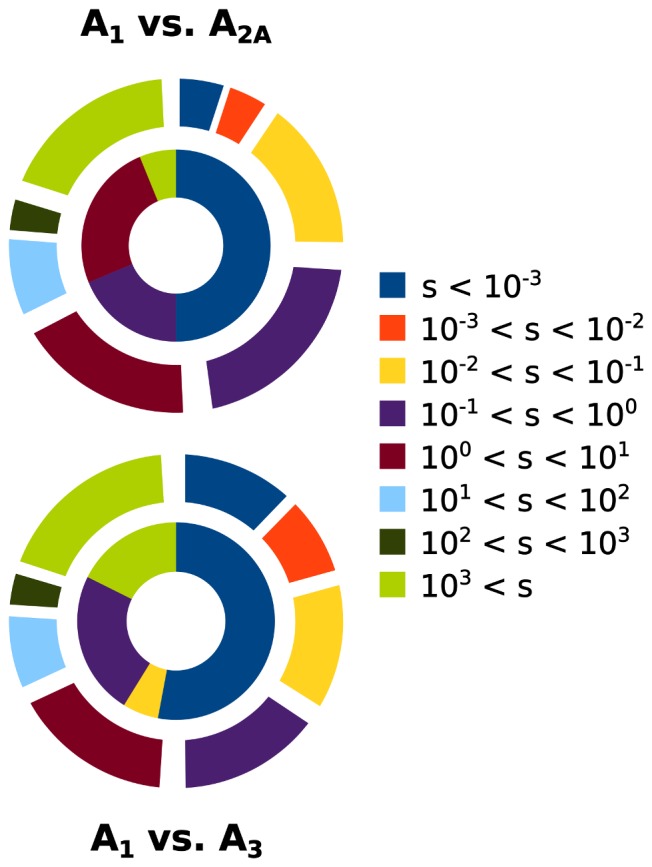
Comparing the selectivity of ligands from this work with ChEMBL data. Selectivity statistics for experimentally measured affinities of molecules from the ChEMBL database (outer shell) and our screen (inner donut). Selectivity ratios have been binned into log-sized bins, ranging from more than 1000-fold selectivity in either direction to 1.

**Figure 4:**
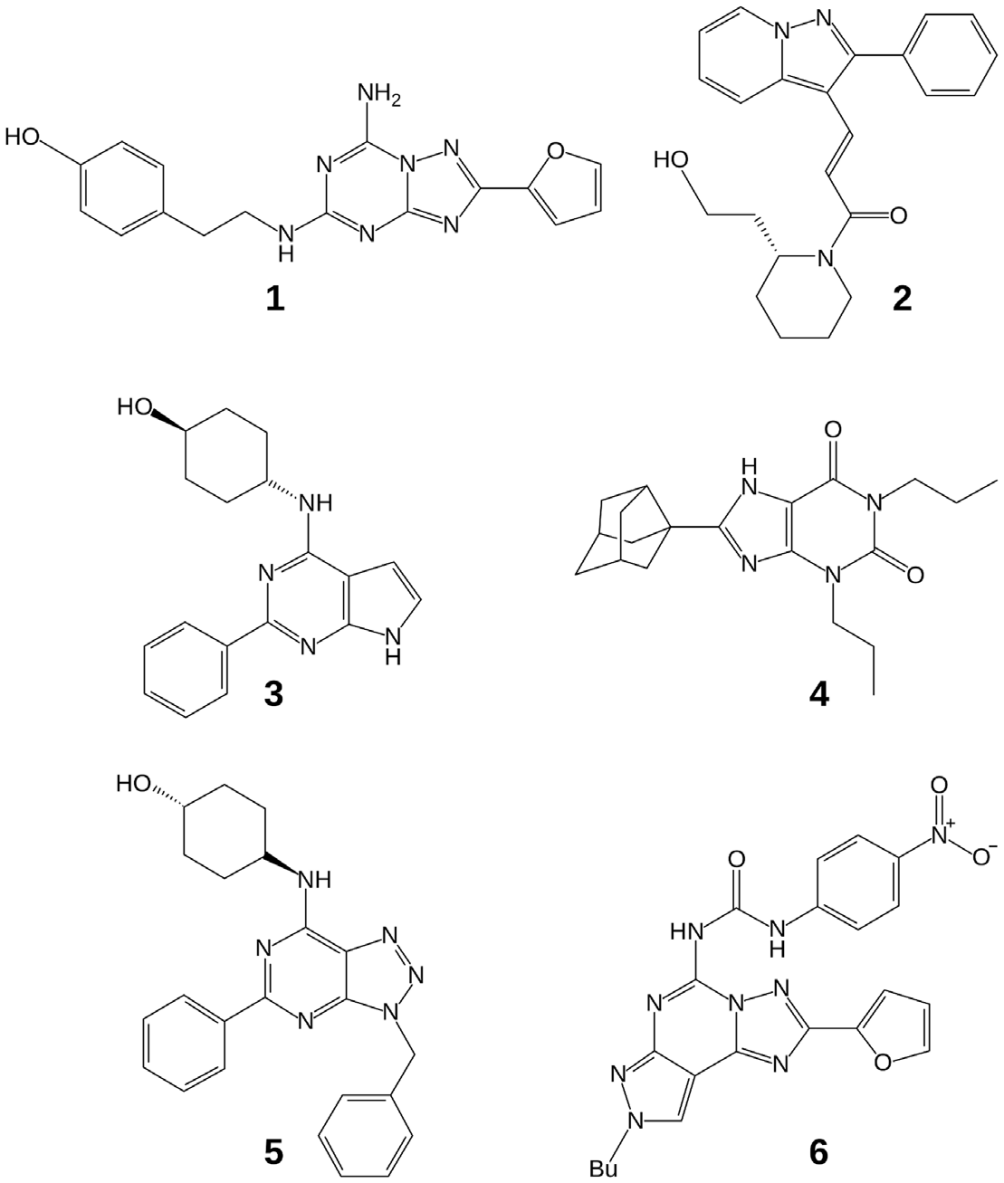
Chart 1. Reference compounds (known selective A_1_AR antagonists) mentioned in the text. K_i_ values are as follows, with targets other than human A_1_AR in parentheses: **1**: K_i_ 0.8 nM [8]; **2**: K_i_ 18 nM; **3**: K_i_ 1 nM; **4**: K_i_ 1 nM [11]; **5**: K_i_ 3 nM (bovine A_1_AR [20]); **6**: K_i_ 584 nM [21].

**Figure 5:**
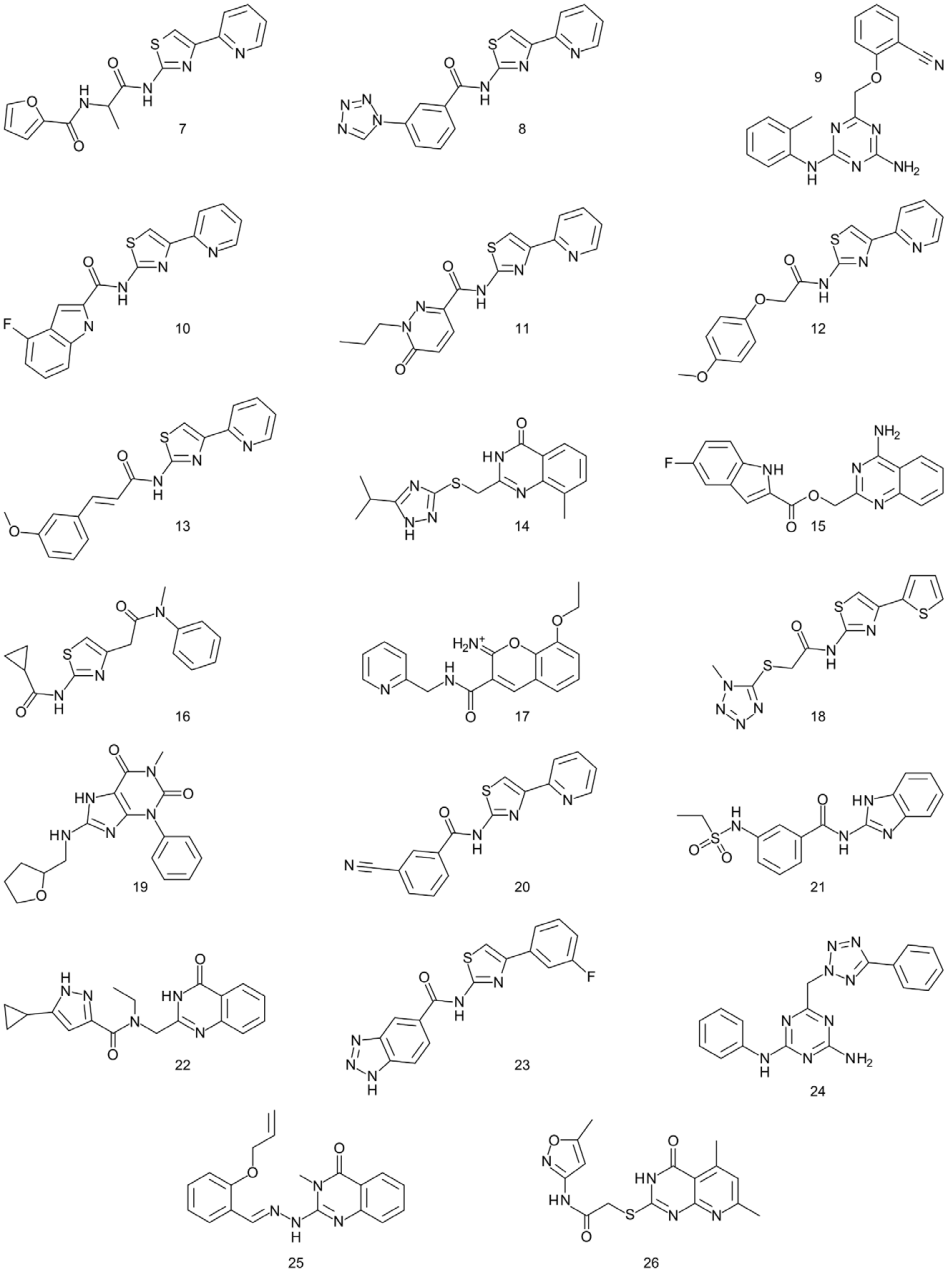
Chart 2. Molecules identified in this study. Binding affinity data at three AR subtypes are presented in Table 1.

The human A_1_AR subtype was investigated in this study because it shares a high level of sequence identity (40%) with the A_2A_AR. It should thus be possible to model the A_1_AR by homology with high confidence. While this homology model was the only three-dimensional structure of a protein employed in the screening, all compounds were also tested in receptor binding assays against two other AR subtypes in order to investigate the intrinsic selectivity of the model.

## Methods

### Homology Modeling

The 3D structure of the A_1_AR was generated with the software MODELLER [16], [17] using the X-ray structure of the A_2A_AR (PDB 3EML; the only structure available at the time) [8] as a template. The overall sequence identity between the two proteins is 40%, with an additional 21% similar residues. Since the A_2A_AR structure was solved with the antagonist **1**, water molecules, and stearic acid, these heteroatoms were included during A_1_AR model building to obtain a model conformation closer to the A_2A_AR X-ray structure.

Due to the stochastic conformational sampling used for homology modeling, an ensemble of 100 models was constructed using the same alignment. The most accurate model from this ensemble of models was selected according to the DOPE (Discrete Optimized Protein Energy) atomic distance-dependent statistical potential function [18], which is included in MODELLER. However, because DOPE had only been trained and tested on globular proteins, its usefulness for assessing models of membrane proteins such as GPCRs was unclear. Thus, globular regions were extracted from the modeled A_1_AR structures by selecting residues in a 6 Å sphere around C7, C11, and C12 of **1**. This extraction resulted in 100 approximately globular protein fragments. These fragments were scored with DOPE and DOPE_HR (DOPE high resolution) and the top five scoring models were inspected visually. Criteria in this visual inspection were the absence of obvious steric clashes with **1**, the absence of kinks in the helices, an orientation of the sidechain of Asn254^6.55^ away from the main chain, and preservation of the disulfide bonds between Cys80^3.25^-Cys169 and Cys260^6.61^-Cys263^7.28^ (superscripts denote Ballesteros-Weinstein numbers [19]). The model that was chosen among the top five according to these criteria was denoted as model **O**.

### Model Refinement

As shown previously, adapting the orthosteric sites of GPCR homology models to known ligands improves pose fidelity and hit rates [20]. Thus, for optimization of model **O**, binding site residues within a 6 Å radius around the equivalent position of **1** (the ligand in 3EML) were iteratively refined with CHARMM [21] and MODELLER. The residues selected for optimization were also compared to mutagenesis studies of the A_1_AR in recognition of agonists and antagonists [22], [23]. Residues that caused major changes in binding affinity (up to 100-fold decrease) after alanine substitution were checked against the selection of residues within 6 Å of the ligand. In all cases, the residues that contributed to a loss of binding affinity after alanine substitution were included in the selection.

For the part of the refinement using CHARMM, the CHARMm22 force field (Accelrys, Inc.) was used, and harmonic restraints with a force constant of 50 kcal/mol·Å^2^ and a minimum at 2.4 Å were assigned to the hydrogen bonds formed between the respective ligand and Asn254^6.55^, the key recognition residue in the A_1_AR binding pocket. A known ligand of the A_1_AR (4-[(3-benzyl-5-phenyl-triazolo[4,5-e]pyrimidin-7-yl)amino]cyclohexan-1-ol; **5**, [24]) was placed manually in the binding site (to ensure correct orientation, i.e. maintenance of the two hydrogen bonds with Asn254^6.55^) and force-field minimized while keeping the adjacent residues fixed. The optimized ligand pose was then included in the following re-modeling step with MODELLER. This procedure of force-field minimizing the ligand and remodeling with MODELLER was repeated until the atomic positions of the active site residues and the ligand converged. To check for bias introduced by the optimization with the reference triazolo-pyrimidine derivative **5**, a second AR antagonist (1-(8-butyl-2-furan-2-yl-8H-pyrazolo[4,3-e] [1], [2], [4]triazolo[1,5-c]pyrimidin-5-yl)-3-(4-nitro-phenyl)-urea, **6** [25]) was manually placed in the binding site, again making making sure that the hydrogen bonds with Asn254^6.55^ are formed, and minimized with PLOP [26], [27]. Residues whose interaction with the ligand had unfavorable force field energy values (Ala66^2.61^, Ile69^2.64^, Phe171, Leu250^6.51^, and Ile274^7.39^) were sidechain-optimized followed by minimization together with the ligand. Both ligands had been part of a set of 3276 A_1_AR binders extracted from the WOMBAT database [28]. They were selected for the refinement process because they docked in poses interacting with Asn254^6.55^ and ranked highly when docked to model **O**. The final refined structure, termed model **A**, was used in the first docking round (see below and Fig. 1A).

Using the ligand data acquired in round one, the orthosteric binding site of the A_1_AR was optimized a second time. In this round of refinement (resulting in model **B**; Fig. 1B), residues were chosen based on their deviation from the corresponding residues in the A_2A_AR structure. In particular, extracellular loop 3 (ECL3; residues Phe259^6.60^ to Cys263^7.28^) and adjacent residues in helix 7 (up to Ser267^7.32^) were rebuilt to maintain the salt bridge between His264^7.29^ and Glu172. Moreover, the “toggle switch” Trp247^6.48^ and the adjacent His251^6.52^, which showed large deviations of up to 140° in their χ_1_ angles, were manually flipped and then minimized. No restraints were applied during loop rebuilding, but all loop conformations that did not place the C_α_ and C_γ_ atoms of His264^7.29^ within 0.8 Å of the equivalent positions of these atoms in the A_2A_AR structure 3EML were discarded. The sidechain orientations for all other residues were sampled and minimized together with **8** (Figure 5), the ligand that was used in this refinement. All optimizations in this and the third round were done with PLOP and the pose of **8** was the one obtained from docking.

Refinements in the third round again used the most selective ligand identified in the previous rounds (**8**) and optimized the sidechains of the same residues as before. Multiple structures were generated, clustered by sidechain conformations and assessed by calculating their ability to rank the ligands over the decoys of rounds one and two (assessed via the value of the area under the curve [logAUC] of receiver-operator characteristic [logROC] plots). For each sidechain conformation cluster, the best structure according to the logAUC criterion was kept and used in docking (models **C** and **D**; Fig. 1C and 1D, respectively).

### Docking

All calculations were carried out using DOCK3.5.54 [29]–[32] and the approximately 2.2 M molecules of ZINC’s lead-like subset [33]. The molecules in this subset are between 250 and 350 g/mol in molecular weight, have less than 7 rotatable bonds, and have an xlogP between 2.5 and 3.5.

The docking spheres used as anchor points in the binding site to position the database molecules in the orthosteric pocket were calculated based on the heavy-atom positions of carazolol and **1** when superimposing the backbone atoms of the β_2_-adrenergic receptor (PDB code 2RH1) and A_2A_AR, respectively, with the A_1_AR model. Where necessary, spheres were moved manually to obtain a more homogenous distribution. During docking, every molecule was fitted onto spheres chosen by the algorithm based on the similarity of the distances between the spheres and corresponding heavy atoms in the molecules. Each molecule pose was minimized for 25 steps with the simplex method. Finally, the binding affinity was estimated by adding the electrostatic and van der Waals interaction energies and correcting for solvation penalty. These energy terms were obtained from precalculated values stored on cubic grids. To emphasize the highly conserved interaction of adenosine with Asn254^6.55^, partial charges on the polar side chain atoms were amplified by 0.4 units in such a way that the overall charge of the residue remained neutral. After docking, the top 500 poses were inspected visually to filter out molecules with unsatisfied hydrogen bond donors or acceptors, incorrect protonation states, unlikely binding modes due to incorrect parametrization, or highly strained conformations. The selected molecules were acquired from their respective vendors as listed in the ZINC database.

### Selectivity Ratios of known AR Ligands

All ligands annotated with an activity value against at least one of the investigated AR subtypes were downloaded from version 12 of the ChEMBL database [34]. The data was made uniform by keeping only affinities measured as K_i_. K_i_-values described as “greater than” a threshold (ranging from 10^−8 ^M to 10^−2^ M, depending on the study the data originated from) were treated as “inactive”. For molecules with more than one independently measured K_i_ value, the average was calculated. Cases with at least one “active” and one “inactive”, i.e. inconsistent, classification with respect to a particular receptor were discarded. The selectivity ratio was calculated by dividing the respective K_i_ values of one ligand against two different receptor subtypes, and binned according to their ratio. The K_i_-value of an inactive molecule was arbitrarily set to 1 M, except for cases where a molecule was inactive against both investigated subtypes, and was thus not considered further in the analysis. The choice of the numerical value for inactive compounds had no influence on the conclusions drawn, as we only compared data that had been obtained with the same settings.

### Experimental Assays

Binding affinity for three human AR (hAR) subtypes was measured using standard radioligand assays and membrane preparations from Chinese hamster ovary (CHO) cells [35] (A_1_ and A_3_) or human embryonic kidney (HEK) 293 cells (A_2A_) stably expressing a hAR subtype (Table 1).

*Receptor binding assays:* [^3^H]8-Cyclopentyl-1,3-dipropylxanthine ([^3^H]DPCPX, 120 Ci/mmol) and [^125^I]*N* ^6^-(4-amino-3-iodobenzyl)adenosine-5'-*N*-methyluronamide ([^125^I]I-AB-MECA, 2200 Ci/mmol) were purchased from Perkin–Elmer Life and Analytical Science (Boston, MA). [^3^H](2-[p-(2-Carboxyethyl)phenyl-ethylamino]-5'-*N*-ethylcarboxamido-adenosine) ([^3^H]CGS21680, 39 Ci/mmol) was purchased from American Radiolabeled Chemicals, Inc. (St. Louis, MO). Other pharmacological reagents were purchased from Tocris-R&D Systems, Inc. (Minneapolis, MN). Test compounds were prepared as 5 mM stock solutions in DMSO and stored frozen.

*Cell Culture and Membrane Preparation:* CHO cells stably expressing the recombinant hA_1_ and hA_3_ARs, and HEK-293 cells stably expressing the hA_2A_AR were cultured in Dulbecco’s modified Eagle medium (DMEM) and F12 (1∶1) supplemented with 10% fetal bovine serum, 100 units/mL penicillin, 100 µg/mL streptomycin, and 2 µmol/mL glutamine. In addition, 800 µg/mL geneticin was added to the A_2A_ media, while 500 µg/mL hygromycin was added to the A_1_ and A_3_ media. After harvesting, cells were homogenized and suspended in PBS. Cells were then centrifuged at 240 *g* for 5 min, and the pellet was resuspended in 50 mM Tris-HCl buffer (pH 7.5) containing 10 mM MgCl_2_. The suspension was homogenized and was then ultra-centrifuged at 14,330 *g* for 30 min at 4°C. The resultant pellets were resuspended in Tris buffer and incubated with adenosine deaminase (3 units/mL) for 30 min at 37°C. The suspension was homogenized with an electric homogenizer for 10 sec, pipetted into 1 mL vials and then stored at -80°C until the binding experiments. The protein concentration was measured using the BCA Protein Assay Kit from Pierce Biotechnology, Inc. (Rockford, IL) [36].

*Binding assays:* Standard radioligand binding assays for A_1_, A_2A_, and A_3_ARs were used [37]–[39]. Into each tube in the binding assay was added 50 µL of increasing concentrations of the test ligand in Tris-HCl buffer (50 mM, pH 7.5) containing 10 mM MgCl_2_, 50 µL of the appropriate agonist radioligand, and finally 100 µL of membrane suspension. For the A_1_AR (22 µg of protein/tube) the radioligand used was [^3^H]DPCPX (final concentration of 0.5 nM). For the A_2A_AR (20 µg/tube) the radioligand used was [^3^H]CGS21680 (final concentration 10 nM). For the A_3_AR (21 µg/tube) the radioligand used was [^125^I]I-AB-MECA (final concentration 0.2 nM). Nonspecific binding was determined using a final concentration of 10 µM NECA diluted with the buffer. The mixtures were incubated at 25°C for 60 min in a shaking water bath. Binding reactions were terminated by filtration through Brandel GF/B filters under a reduced pressure using a M-24 cell harvester (Brandel, Gaithersburg, MD). Filters were washed three times with 3 mL of 50 mM ice-cold Tris-HCl buffer (pH 7.5). Filters for A_1_ and A_2A_AR binding were placed in scintillation vials containing 5 mL of Hydrofluor scintillation buffer and counted using a Perkin Elmer Liquid Scintillation Analyzer (Tri-Carb 2810TR). Filters for A_3_AR binding were counted using a Packard Cobra II γ-counter.

*Data analysis:* Binding and functional parameters were calculated using Prism 5.0 software (GraphPAD, San Diego, CA, USA). IC_50_ values obtained from binding inhibition curves were converted to K_i_ values using the Cheng-Prusoff equation [40]. Data were expressed as mean ± standard error or percentage inhibition at 10 µM.

## Results

### Model Building & Docking

In total, four conformational variants of the A_1_AR homology model were used during docking and ligand selection (Fig. 1). Model **A** was the original model, refined with the two previously known ligands **5** and **6**; model **B** was obtained by rebuilding ECL3 and adjacent residues around ligand **8**; and models **C** and **D** were generated by further adapting the binding site to the most selective ligand previously identified in this study (**8**; binding mode shown in Fig. 2) using logAUC and side chain orientation diversity as model selection criteria. In terms of heavy-atom RMSD, models **C** and **D** differed by less than 0.18 Å overall and by less than 1.17 Å in the refined residues in the binding site (Fig. 1). Docked compounds that ranked highly in at least one of the models (Figure 5 and Table S1) were selected after visual inspection and tested experimentally for receptor affinity. These diverse compounds included thiazole (**7**, **8**, **10–13**, **16**, **18**, **20**, and **23**), 1,3,5-triazine (**9** and **24**) and other heterocyclic cores. Thiazoles and 1,2,4-triazines are known chemotypes for binding to ARs [41], [42]. A xanthine derivative **19**, unusual in its 1-phenyl substitution, also appeared as a hit. According to the docking predictions, this phenyl ring of **19** was oriented away from Asn254^6.55^ toward the pocket lined by Val62^2.57^, Ala66^2.61^, and Val87^3.32^. A commonality of all compounds was that they form two hydrogen bonds with Asn254^6.55^ in the calculated poses.

Table 1 lists all ligands that inhibited radioligand binding to at least one hAR subtype by more than 50% at a concentration of 10 µM and were thus classified as active. Their two-dimensional structures are shown in Figure 5. Data for molecules that did not pass this threshold are presented in Table S1. Table 2 lists the total number of molecules tested in each round. In total, we found 8 ligands for the A_1_AR, 15 for the A_2A_AR and 14 for the A_3_AR. The structurally most similar known AR ligand from ChEMBL for each hit, as determined by ECFP4 Tanimoto similarity, is listed in Table S2. One of the ligands (**14**) may be regarded as a novel AR ligand because its Tanimoto similarity to the most similar known ligand is less than 0.26, which is generally accepted as a strict cutoff [43]. By a more relaxed cutoff of 0.4 [44], five more compounds (**15**, **21**, **22**, **25**, **26**) are novel. Table 2 furthermore details the performance of the individual models by their ability to predict ligands. Model **C** was the most unproductive, having no correct ligand predictions. It is interesting to note that there is no clear trend in the performance in terms of selectivity. One could have assumed that models productive for one AR subtype might perform badly in retrieving ligands for a different one (despite all of them being models with the A_1_AR sequence). This only seems to be the case for model **A** (retrieving more A_2A_ and A_3_AR ligands than A_1_AR ligands), but not the other ones, which tend to find approximately equal numbers for ligands of all subtypes.

### Selectivity Calculations

A total of 2181 ligands from the ChEMBL database had experimentally determined non-negative K_i_ values against both A_1_ and A_2A_, and 1476 molecules had such measurements against A_1_ and A_3_. Only 77 of all known experimental AR ligands had ambiguous classifications as being “inactive” and “active” against at least one receptor, and were thus not investigated further. The results are presented as pie charts in Fig. 3. Subtype-selective molecules were slightly more prevalent between A_1_ and A_3_ than between A_1_ and A_2A_: 66% and 58% of the ligands were more than 10-fold selective in either direction, respectively. The ligands emerging from this screen tended to be more selective for A_2A_ and A_3_ than A_1_, as can be seen from the larger areas for the corresponding selectivity ratios (inner donuts in Fig. 3). Although the numbers have to be viewed with caution because of the limitations of statistics of small numbers, these observations contrast those for the ChEMBL ligands, which tended to be more selective for A_1_.

## Discussion

Three main results emerge from this study. First, as has been shown previously [45], [46], different models (or X-ray structures) of the same receptor yield different ligand sets, even when screening the same diverse library. Interestingly, the performance of the various models, both in absolute number of actual ligands as well as in terms of selectivity, differed widely. This fact is both en- and discouraging. It is encouraging, because it means that even using models with large structural deviations from a closely related template (i.e. the conformation of ECL3, the lack of the conserved salt bridge between His264^7.29^ and Glu172, and the orientation of Trp247^6.48^) such as model **A**, docking is likely to find pharmacologically validated ligands. Conversely, it is discouraging, as the presumably refined model **C** did not yield any ligands. This is particularly striking considering the small differences between models **C** and **D**. We did not exclude the molecules tested in earlier rounds of screening during the subsequent ones, yet the vast majority of ligands identified in one model did not appear in the top ranks of a screen against another one (data not shown). Such behavior is a testament to the conformational flexibility of GPCRs, but also to the sensitivity of docking to small changes in the protein structure. In combination, it can be exploited to identify larger numbers of ligands by docking to more than one protein conformation. Any model of a protein structure (including the X-ray solution) represents only one possibility from the continuum of conformations. Thus, using differently optimized models (e.g. obtained by slightly different ligand placements or different force field parameters), the set of identified ligands would have changed. Yet, the overall performance, with some models being able to recognize ligands and some not, would be similar. This fact might also be considered disheartening for approaches that aim to include receptor flexibility via docking to multiple conformations of a receptor and calculating the average rank of a molecule across all structures.

Second, docking to GPCRs, even using “only” homology models, works well. The screen against the A_1_AR was successful by all criteria, with a hit rate of 21% and potent compounds with K_i_ values as low as 400 nM for the 2*H*-chromen-2-imine derivative **17**. Some of the ligands also represent novel chemotypes for the A_1_AR, such as **17** and related, albeit only weakly active, derivatives quinazolin-4(3*H*)-ones (**14**, **22**, **25**) and a pyrido[2,3-*d*]pyrimidin-4(3*H*)-one (**26**). In particular, the ligands identified with model **D** tend to have ECFP4 Tanimoto similarity values of less than 0.40 when compared to the 7173 AR ligands in the ChEMBL database. The reason for the relatively few genuinely novel ligands presumably lies in the bias of the library, as shown before [47]. However, the overall performance of this screen is in line with previous docking studies that identified numerous and potent GPCR ligands [9], [45]–[49]. As was the case here, most of these campaigns targeted a Class A GPCR that binds small organic molecules. Such receptors tend to have rather narrow, well-defined binding sites – in contrast to the CXCR4 receptor, the only peptide-bound GPCR structure elucidated so far [50]. Smaller binding pockets make for narrower physical search spaces which is likely one of the reasons behind the tractability of these GPCRs by docking and similar approaches.

Third, for receptors with high degrees of similarity, such as the ARs, selective compounds cannot be predicted solely by docking to one receptor subtype. Most of the ligands identified as A_1_AR hits also bound to one of the other AR subtypes, and *vice versa*. In fact, the screen directed toward the A_1_AR worked even better against the A_3_AR, with a hit rate of 36% and the most potent compound inhibiting with a K_i_ of 36 nM. This is an advantage if it is desired to discover ligands for other related GPCR subtypes within a single screening process.

However, there is one compound, **8**, which appears selective for the A_1_AR by the criteria used in this screen. In addition, some of the ligands were also moderately selective in binding to the A_3_AR, which may be due to the fact that the binding pocket of the A_3_AR is the most divergent one when comparing the three AR subtypes (Table S3), suggesting the relative ease of achieving A_3_AR selectivity. This tendency to cross over to other subtypes in the screening process can be expected from the similarity of the binding sites. It is difficult to estimate, however, to what degree the use of homology models affected the selectivity of the compounds. Bias stemming from the template used (the A_2A_AR) cannot be ruled out, but cannot be the only factor as evidenced by the many compounds binding to A_3_AR. Very likely, even computational screens employing X-ray structures result in similarly nonsubtype-selective hit compounds. However, because biochemical testing is limited to the targeted subtype in most studies, this does not become apparent. As a further example of this observation, in the A_2A_AR screen by Carlsson et al. [10], which is based on a crystal structure, several ligands were found that had mixed selectivity for the A_2A_ and A_3_ARs.

Docking will undoubtedly continue to play a significant role in the quest for novel GPCR ligands, as it has been able to consistently identify potent and chemically novel ligands for a variety of receptors. The targeted identification of *selective* compounds by combining multiple approaches to model the same receptor and closely related members of the same protein family will be the topic of future investigations. Furthermore, the most promising hits from this study, such as a mixed A_1_/A_2A_AR ligand, i.e. the 2*H*-chromen-2-imine derivative **17**, or a moderately potent and slightly selective A_3_AR ligand, i.e. 1,3,5-triazine derivative **24**, could now be optimized structurally for AR affinity and selectivity.

## Supporting Information

Table S1 Ligands that were tested and replaced less than 50% of radioligand at 10 µM in all targets. **n = 2.
(PDF)

Table S2 **Compounds in ChEMBL most similar to the ligands identified in this study.**
(PDF)

Table S3 **Comparison of binding site residues between A_1_AR, A_2A_AR and A_3_AR. ^a^superscripts give the Ballesteros-Weinstein numbers.**
(PDF)

## References

[1] Overington JP, Al-Lazikani B, Hopkins AL (2006) How many drug targets are there? Nat Rev Drug Discov 5: 993–995. 17139284 10.1038/nrd2199

[2] Rosenbaum DM, Rasmussen SG, Kobilka BK (2009) The structure and function of G-protein-coupled receptors. Nature 459: 356–363. 19458711 10.1038/nature08144 PMC3967846

[3] Kolb P, Klebe G (2011) The golden age of GPCR structural biology: any impact on drug design? Angew Chem Int Ed 50: 11573–11575. 10.1002/anie.201105869 22052652

[4] Shoichet BK, Kobilka B (2012) Structure-based drug screening for G-protein-coupled receptors. Trends Pharmacol Sci 33: 268–272. 22503476 10.1016/j.tips.2012.03.007 PMC3523194

[5] Kolb P, Ferreira RS, Irwin JJ, Shoichet BK (2009) Docking and chemoinformatic screens for new ligands and targets. Curr Opin Biotechnol 20: 429–436. 19733475 10.1016/j.copbio.2009.08.003 PMC2766606

[6] Jacobson KA, Costanzi S (2012) New insights for drug design from the X-ray crystallographic structures of GPCRs. Mol Pharmacol 82: 361–371. 22695719 10.1124/mol.112.079335 PMC3422707

[7] Peeters MC, van Westen GJP, Li Q, IJzerman AP (2011) Importance of the extracellular loops in G protein-coupled receptors for ligand recognition and receptor activation. Trends Pharmacol Sci 32: 35–42. 21075459 10.1016/j.tips.2010.10.001

[8] Jaakola VP, Griffith MT, Hanson MA, Cherezov V, Chien EYT, et al (2008) The 2.6 Å Crystal Structure of a Human A_2A_ Adenosine Receptor Bound to an Antagonist. Science 322: 1211–1217. 18832607 10.1126/science.1164772 PMC2586971

[9] Ongini E, Dionisotti S, Gessi S, Irenius E, Fredholm BB (1999) Comparison of CGS 15943, ZM 241385 and SCH 58261 as antagonists at human adenosine receptors. Naunyn Schmiedebergs Arch Pharmacol 359: 7–10. 9933143 10.1007/pl00005326

[10] Carlsson J, Yoo L, Gao ZG, Irwin JJ, Shoichet BK, et al (2010) Structure-Based Discovery of A_2A_ Adenosine Receptor Ligands. J Med Chem 53: 3748–3755. 20405927 10.1021/jm100240h PMC2865168

[11] Jacobson K, Gao Z (2006) Adenosine receptors as therapeutic targets. Nat Rev Drug Discov 5: 247–264. 16518376 10.1038/nrd1983 PMC3463109

[12] Mueller CE, Jacobson KA (2011) Recent developments in adenosine receptor ligands and their potential as novel drugs. Biochim Biophys Acta 1808: 1290–1308. 21185259 10.1016/j.bbamem.2010.12.017 PMC3437328

[13] Terai T, Kusunoki T, Kita Y, Nakano K, Nishina N, et al (1996) Protective effects of FK453, a potent nonxanthine adenosine A1 receptor antagonist, on glycerol-induced acute renal failure in rats. Drug Dev Res 39: 47–53.

[14] Mitrovic V, Seferovic P, Dodic S, Krotin M, Neskovic A, et al (2009) Cardio-renal effects of the A1 adenosine receptor antagonist SLV320 in patients with heart failure. Circ Heart Fail 2: 523–531. 19919976 10.1161/CIRCHEARTFAILURE.108.798389

[15] Slawski MT, Givertz MM (2009) Rolofylline: a selective adenosine 1 receptor antagonist for the treatment of heart failure. Exp Opin Pharmacother 10: 311–322. 10.1517/14656560802682213 19236201

[16] Sali A, Blundell TL (1993) Comparative protein modelling by satisfaction of spatial restraints. J Mol Biol 234: 779–815. 8254673 10.1006/jmbi.1993.1626

[17] Fiser A, Do RKG, Sali A (2000) Modeling of loops in protein structures. Protein Sci 9: 1753–1773. 11045621 10.1110/ps.9.9.1753 PMC2144714

[18] Shen MY, Sali A (2006) Statistical potential for assessment and prediction of protein structures. Protein Sci 15: 2507–2524. 17075131 10.1110/ps.062416606 PMC2242414

[19] Ballesteros JA, Weinstein H (1995) Integrated methods for the construction of three dimensional models and computational probing of structure function relations in G protein-coupled receptors. In: Sealfon SC, Conn PM, editors. Methods in Neurosciences. San Diego: Academic Press. 366–428.

[20] Phatak SS, Gatica EA, Cavasotto CN (2010) Ligand-steered modeling and docking: A benchmarking study in class A G-protein-coupled receptors. J Chem Inf Model 50: 2119 2128. 10.1021/ci100285f 21080692

[21] Brooks BR, Bruccoleri RE, Olafson BD, States DJ, Swaminathan S, et al (1983) CHARMM: A program for macromolecular energy, minimization, and dynamics calculations. J Comp Chem 4: 187–217.

[22] Fredholm BB, IJzerman AP, Jacobson KA, Klotz KN, Linden J (2001) International Union of Pharmacology. XXV. Nomenclature and classification of adenosine receptors. Pharm Rev 53: 527–552. 11734617 PMC9389454

[23] Rivkees SA, Barbhaiya H, IJzerman AP (1999) Identification of the adenine binding site of the human A_1_ adenosine receptor. J Biol Chem 274: 3617–3621. 9920910 10.1074/jbc.274.6.3617

[24] Massarelli I, Coi A, Pietra D, Nofal FA, Biagi G, et al (2008) QSAR study on a novel series of 8-azaadenine analogues proposed as A1 adenosine receptor antagonists. Eur J Med Chem 43: 114–121. 17418915 10.1016/j.ejmech.2007.02.009

[25] Baraldi PG, Cacciari B, Moro S, Spalluto G, Pastorin G, et al (2002) Synthesis, biological activity, and molecular modeling investigation of new pyrazolo[4,3-e]-1,2,4-triazolo[1,5-c]pyrimidine derivatives as human A3 adenosine receptor antagonists. J Med Chem 45: 770–780. 11831890 10.1021/jm0109614

[26] Jacobson MP, Kaminski GA, Friesner RA, Rapp CS (2002) Force Field Validation Using Protein Sidechain Prediction. J Phys Chem B 106: 11673–11680.

[27] Jacobson MP, Pincus DL, Rapp CS, Day TJF, Honig B, et al (2004) A Hierarchical Approach to All-Atom Loop Prediction. Proteins 55: 351–367. 15048827 10.1002/prot.10613

[28] Olah M, Mracec M, Ostopovici L, Rad R, Bora A, et al.. (2004) WOMBAT: World of Molecular Bioactivity. In: Oprea TI, editor. Chemoinformatics in Drug Discovery. New York: Wiley-VCH. 223–239.

[29] Kuntz ID, Blaney JM, Oatley SJ, Langridge R, Ferrin TE (1982) A geometric approach to macromolecule-ligand interactions. J Mol Biol 161: 269–288. 7154081 10.1016/0022-2836(82)90153-x

[30] Meng EC, Shoichet BK, Kuntz ID (1992) Automated docking with grid-based energy evaluation. J Comput Chem 13: 505–524.

[31] Shoichet BK, Kuntz ID (1993) Matching chemistry and shape in molecular docking. Protein Eng 6: 723–732. 7504257 10.1093/protein/6.7.723

[32] Shoichet BK, Leach AR, Kuntz ID (1999) Ligand solvation in molecular docking. Proteins Struct Funct Genet 34: 4–16. 10336382 10.1002/(sici)1097-0134(19990101)34:1<4::aid-prot2>3.0.co;2-6

[33] Irwin JJ, Shoichet BK (2005) ZINC: a free database of commercially available compounds for virtual screening. J Chem Inf Model 45: 177–182. 15667143 10.1021/ci049714 PMC1360656

[34] Gaulton A, Bellis LJ, Bento AP, Chambers J, Davies M, et al (2012) ChEMBL: a large-scale bioactivity database for drug discovery. Nucleic Acids Res 40: D1100–D1107. 21948594 10.1093/nar/gkr777 PMC3245175

[35] Klotz KN, Hessling J, Hegler J, Owman C, Kull B, et al (1998) Comparative pharmacology of human adenosine receptor subtypes - characterization of stably transfected receptors in CHO cells. Naunyn-Schmiedeberg’s Arch Pharmacol 357: 1–9. 9459566 10.1007/pl00005131

[36] Bradford MM (1976) A rapid and sensitive method for the quantitation of microgram quantities of protein utilizing the principle of protein-dye binding. Anal Biochem 72: 248–254. 942051 10.1016/0003-2697(76)90527-3

[37] Klotz KN, Lohse MJ, Schwabe U, Cristalli G, Vittori S, et al (1989) 2-Chloro-N^6^-[^3^H]cyclopentyladenosine ([^3^H]CCPA)-a high affinity agonist radioligand for A_1_ adenosine receptors. Naunyn Schmiedebergs Arch Pharmacol 340: 679–683. 2615857 10.1007/BF00717744

[38] Jarvis MF, Schutz R, Hutchison AJ, Do E, Sills MA, et al (1989) [^3^H]CGS 21680, a selective A_2_ adenosine receptor agonist directly labels A_2_ receptors in rat brain. J Pharmacol Exp Ther 251: 888–893. 2600819

[39] Olah ME, Gallo-Rodriguez C, Jacobson KA, Stiles GL (1994) ^125^I-4-aminobenzyl-5’-N-methylcarboxamidoadenosine, a high affinity radioligand for the rat A_3_ adenosine receptor. Mol Pharmacol 45: 978–982. 8190112 PMC5553074

[40] Cheng YC, Prusoff HR (1973) Relationship between the inhibition constant (K_i_) and the concentration of inhibitor which causes 50 per cent inhibition (I50) of an enzymatic reaction. Biochem Pharmacol 22: 3099–3108. 4202581 10.1016/0006-2952(73)90196-2

[41] Baraldi PG, Preti D, Borea PA, Varani K (2012) Medicinal chemistry of A_3_ Adenosine Receptor modulators: pharmacological activities and therapeutic implications. J Med Chem 55: 5676–5703. 22468757 10.1021/jm300087j

[42] Congreve M, Andrews SP, Doré AS, Hollenstein K, Hurrell E, et al (2012) Discovery of 1,2,4-triazine derivatives as Adenosine A_2A_ antagonists using structure based drug design. J Med Chem 55: 1898–1903. 22220592 10.1021/jm201376w PMC3308197

[43] Steffen A, Kogej T, Tyrchan C, Engkvist O (2009) Comparison of molecular fingerprint methods on the basis of biological profile data. J Chem Inf Model 49: 338–347. 19434835 10.1021/ci800326z

[44] Wawer M, Bajorath J (2010) Similarity-potency trees: a method to search for SAR information in compound data sets and derive SAR rules. J Chem Inf Model 50: 1395–1409. 20726598 10.1021/ci100197b

[45] Carlsson J, Coleman RG, Setola V, Irwin JJ, Fan H, et al (2011) Ligand discovery from a dopamine D_3_ receptor homology model and crystal structure. Nat Chem Biol 7: 769–778. 21926995 10.1038/nchembio.662 PMC3197762

[46] Mysinger MM, Weiss DR, Ziarek JJ, Gravel S, Doak AK, et al (2012) Structure-based ligand discovery for the protein-protein interface of chemokine receptor CXCR4. Proc Natl Acad Sci USA 109: 5517–5522. 22431600 10.1073/pnas.1120431109 PMC3325704

[47] Kolb P, Rosenbaum DM, Irwin JJ, Fung JJ, Kobilka BK, et al (2009) Structure-based discovery of β_2_-adrenergic receptor ligands. Proc Natl Acad Sci USA 106: 6843–6848. 19342484 10.1073/pnas.0812657106 PMC2672528

[48] Sabio M, Jones K, Topiol S (2008) Use of the X-ray structure of the β_2_-adrenergic receptor for drug discovery. Part 2: Identification of active compounds. Bioorg Med Chem Lett 18: 5391–5395. 18829308 10.1016/j.bmcl.2008.09.046

[49] Katritch V, Jaakola VP, Lane JR, Lin J, IJzerman AP, et al (2010) Structure-Based Discovery of Novel Chemotypes for Adenosine A_2A_ Receptor Antagonists. J Med Chem 53: 1799–1809. 20095623 10.1021/jm901647p PMC2826142

[50] Wu B, Chien EY, Mol CD, Fenalti G, Liu W, et al (2012) Structures of the CXCR4 chemokine GPCR with small-molecule and cyclic peptide antagonists. Science 330: 1066–1071. 10.1126/science.1194396 PMC3074590 20929726

