# Supplementary material for: Limits of Ligand Selectivity from Docking to Models: In Silico Screening for A1 Adenosine Receptor Antagonists
Source: PLoS One. 2012 Nov 21;7(11):e49910. doi: 10.1371/journal.pone.0049910 (PMC3503826; doi:10.1371/journal.pone.0049910)
Supplement: Table S1 — Ligands that were tested and replaced less than 50% of radioligand at 10 µM in all targets. **n = 2. (PDF) [file pone.0049910.s001.pdf]

Table S1: Ligands that were tested and replaced less than 50% of radioligand at 10 μM in all targets.

ZINC ID	SMILES	A_1AR	A_{2A}AR	A_3AR	Model
06844430	CCOC(=O)c1cnc2nc(nn2c1[O-])NCc3cccc(c3)OC	3.4 ± 3.4%	0%	11.1 ± 3.8%	A
08653875	CCOC(=O)Cc1c(nc2cc([nH]n2c1=O)c3ccc(cc3)Cl)C	0%	2.1 ± 2.1%	10.4 ±5.0%	A
05238266	C[C@@H](C(=O)OC(C)C)Sc1nc(c(c(n1)[O-])C#N)c2ccccc2	20.3 ± 20.3%	7.6 ± 4.3%	25.3 ± 6.4%	A
18284742	CCOC(=O)Cc1c([nH]c2cc(nn2c1=O)c3ccc(cc3)C)C	8.9 ± 5.5%	8.1 ± 8.1%	20.7 ± 7.3%	A
18156418	CCOc1ccccc1CNc2nc3nc4c(c(n3n2)[O-])CCC4	10.9 ± 10.9%	8.8 ± 6.3%	26.8 ± 8.1%	A
40488321	Cc1nc(nc(n1)Nc2[nH]c3ccccc3n2)c4ccccc4O	21 ± 2%	37 ± 4%	41 ± 2%	B
08746576	Cn1cccc1C(=O)CSc2n[nH]c3n2c4ccccc4n3	28 ± 6%**	20 ± 4%	38 ± 4%	B
20365432	C[C@@H](c1ccnc2n1nc(n2)NC=[N+](C)C)Oc3cccc(c3)OC	33 ± 3%**	2 ± 2%	19 ± 5%	B
31875387	Cc1c(ccc(n1)C(=O)Nc2nc(cs2)c3ccc(cc3)F)C#N	11 ± 1%**	1 ± 1%	16 ± 7%	B
54203984	Cc1cccc(n1)NC(=O)[C@H]2CCC[NH+](C2)CC(=O)N3CCNC3=O	3 ± 3%	17 ± 5%	28 ± 2%	C
56482974	C[C@H](C(=O)Nc1ccc(c(c1)NC(=O)OC(C)(C)C)F)n2cc[nH+]c2	5 ± 4%	45 ± 5%	53 ± 3%	C
47260884	Cc1cccc(n1)NC(=O)[C@H]2CCC[NH+](C2)CC(=O)NC3CCCC3	4 ± 4%	48 ± 3%	31 ± 6%	C
31584575	CN(Cc1[nH]c(=O)c2ccccc2n1)C(=O)/C=C/c3cccc(c3)C#N	12 ± 7%	12 ± 6%	19 ± 4%	C
43348592	c1ccc(cc1)Oc2ccccc2NC(=O)NC[C@@H](C(F)(F)F)O	3 ± 2%	4 ± 4%	23 ± 6%	C
47260934	Cc1cccc(n1)NC(=O)[C@H]2CCC[NH+](C2)CC(=O)NCC(C)C	10 ± 8%	53 ± 3%	31 ± 2%	C
47260835	CCNC(=O)NC(=O)C[NH+]1CCC[C@@H](C1)C(=O)Nc2cccc(n2)C	8 ± 4%	14 ± 3%	20 ± 5%	C
08724684	Cc1cccc2c1nc([nH]c2=O)/C(=C/c3ccc(c(c3)O)OC)/Cl	10 ± 5%	18 ± 4%	46 ± 5%	D
06647942	c1ccc2c(c1)c(=O)[nH]c(n2)CCC(=O)Nc3[nH]c4ccccc4n3	2 ± 2%	15 ± 4%	13 ± 3%	D
04059122	c1ccc2c(c1)c(=O)cc(o2)C(=O)Nc3nc(cs3)c4ccccn4	4 ± 4%	27 ± 6%	34 ± 2%	D

**n=2
