# Supplementary material for: Limits of Ligand Selectivity from Docking to Models: In Silico Screening for A1 Adenosine Receptor Antagonists
Source: PLoS One. 2012 Nov 21;7(11):e49910. doi: 10.1371/journal.pone.0049910 (PMC3503826; doi:10.1371/journal.pone.0049910)
Supplement: Table S2 — Compounds in ChEMBL most similar to the ligands identified in this study. (PDF) [file pone.0049910.s002.pdf]

Table S2: Compounds in ChEMBL most similar to the ligands identified in this study.

**ID**	**Closest ChEMBL**	sim_T	2D-Structure
**7**	CHEMBL164087	0.53	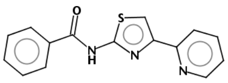
**8**	CHEMBL163040	0.64	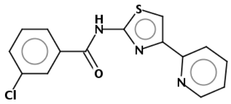
**9**	CHEMBL605584	0.47	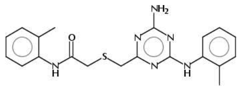
**10**	CHEMBL164087	0.57	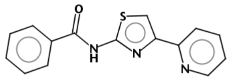
**11**	CHEMBL164087	0.56	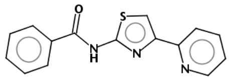
**12**	CHEMBL165784	0.72	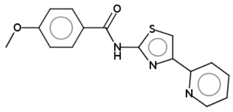
**13**	CHEMBL165784	0.60	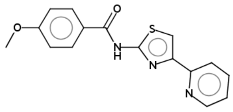
**14**	CHEMBL204780	0.25	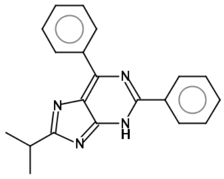
**15**	CHEMBL321441	0.30	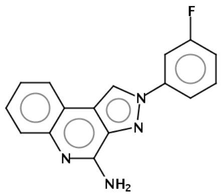
**16**	CHEMBL162808	0.46	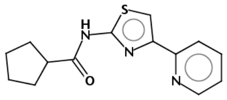
**17**	CHEMBL611964	0.49	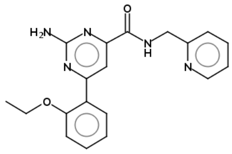
**18**	CHEMBL1650339	0.41	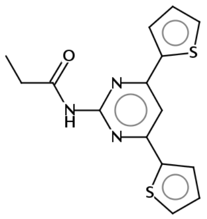

continued on next page

**19**	CHEMBL597167	0.41	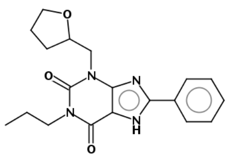
**20**	CHEMBL163040	0.71	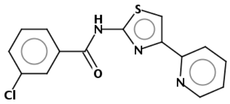
**21**	CHEMBL93600	0.39	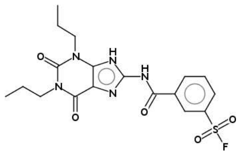
**22**	CHEMBL305516	0.32	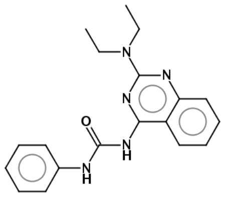
**23**	CHEMBL293437	0.50	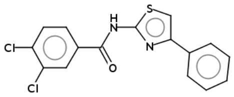
**24**	CHEMBL600404	0.42	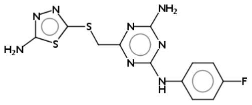
**25**	CHEMBL1097489	0.30	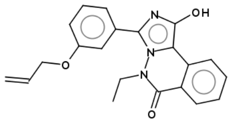
**26**	CHEMBL1098786	0.27	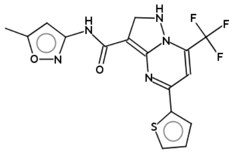
