# Supplementary material for: Limits of Ligand Selectivity from Docking to Models: In Silico Screening for A1 Adenosine Receptor Antagonists
Source: PLoS One. 2012 Nov 21;7(11):e49910. doi: 10.1371/journal.pone.0049910 (PMC3503826; doi:10.1371/journal.pone.0049910)
Supplement: Table S3 — Comparison of binding site residues between A1AR, A2AAR and A3AR. asuperscripts give the Ballesteros-Weinstein numbers. (PDF) [file pone.0049910.s003.pdf]

Table S3: Comparison of binding site residues between A_1AR, $A_{2A}AR$ and A_3AR.

$\mathbf{A_1AR}^a$	$\mathbf{A_{2A}AR}^a$	$\mathbf{A_3AR}^a$
Ile69$^{2.64}$	Ile66$^{2.64}$	Val72$^{2.64}$
Ala84$^{3.29}$	Ala81$^{3.29}$	Thr87$^{3.29}$
Val87$^{3.32}$	Val84$^{3.32}$	Leu90$^{3.32}$
Leu88$^{3.33}$	Leu85$^{3.33}$	Leu91$^{3.33}$
Thr91$^{3.36}$	Thr88$^{3.36}$	Thr94$^{3.36}$
Glu170	Leu167	Gln167
Phe171	Phe168	Phe168
Glu172	Glu169	Val169
Lys173	Asp170	Ser170
Met177	Met174	Met174
Met180$^{5.38}$	Met177$^{5.38}$	Met177$^{5.38}$
Asn184$^{5.42}$	Asn181$^{5.42}$	Ser181$^{5.42}$
Phe185$^{5.43}$	Phe182$^{5.43}$	Phe182$^{5.43}$
Val189$^{5.47}$	Val186$^{5.47}$	Ile186$^{5.47}$
Trp247$^{6.48}$	Trp246$^{6.48}$	Trp243$^{6.48}$
Leu250$^{6.51}$	Leu249$^{6.51}$	Leu246$^{6.51}$
His251$^{6.52}$	His250$^{6.52}$	Ser247$^{6.52}$
Leu253$^{6.54}$	Ile252$^{6.54}$	Ile249$^{6.54}$
Asn254$^{6.55}$	Asn253$^{6.55}$	Asn250$^{6.55}$
Thr257$^{6.58}$	Thr256$^{6.58}$	Ile253$^{6.58}$
His264$^{7.29}$	His264$^{7.29}$	—
Lys265$^{7.30}$	Ala265$^{7.30}$	—
Pro266$^{7.31}$	Pro266$^{7.31}$	Pro260$^{7.31}$
Ser267$^{7.32}$	Leu267$^{7.32}$	Gln261$^{7.32}$
Thr270$^{7.35}$	Met270$^{7.35}$	Leu264$^{7.35}$
Tyr271$^{7.36}$	Tyr271$^{7.36}$	Tyr265$^{7.36}$
Ile274$^{7.39}$	Ile274$^{7.39}$	Ile268$^{7.39}$
Thr277$^{7.42}$	Ser277$^{7.42}$	Ser271$^{7.42}$

[a]superscripts give the Ballesteros-Weinstein numbers
